# Napping Behaviors and Extracurricular Club Activities in Japanese High School Students: Associations with Daytime Sleep Problems

**DOI:** 10.3390/clockssleep1030030

**Published:** 2019-08-09

**Authors:** Koh Mizuno, Kazue Okamoto-Mizuno, Kazuki Iwata

**Affiliations:** 1 Faculty of Education, Tohoku Fukushi University, Sendai 981-8522, Japan; 2 Kansei Fukushi Research Center, Tohoku Fukushi University, Sendai 989-3201, Japan; 3 Faculty of Comprehensive Management, Tohoku Fukushi University, Sendai 981-8522, Japan

**Keywords:** adolescents, athletics, human activities, sleep, students

## Abstract

Although engaging in evening naps and extracurricular activities are popular among Japanese high school students, the associations between these behaviors and daytime sleep problems were unclear. A questionnaire on daily life and sleeping habits was administered to 1314 high school students, aged between 15–17 years. The respondents were categorized by their after-school napping habits (did not nap, napped 1–2 days/week, napped ≥3 days/week), and their extracurricular activities (no activity, cultural club, athletic club). The mean nocturnal sleep duration on weekdays (± standard deviation (SD)) was 390 ± 56 min. This was significantly shorter in those students with a higher number of days/week spent napping (*p* < 0.001), and slightly longer for those in the athletic club (*p* < 0.001). Sleep problems—including subjective insufficient sleep, excessive sleepiness during class ≥3 days/week, and falling asleep during class ≥3 days/week—were reported by 64%, 55%, and 33% of respondents, respectively. A multiple logistic regression analysis showed significantly higher risks for excessive sleepiness among students taking naps ≥3 days/week, and who belonged to athletic clubs. In addition to those factors, students in cultural clubs revealed significantly higher risks for falling asleep during classes. Future studies are required to decrease daytime sleep problems associated with evening naps and extracurricular activities among high school students.

## 1. Introduction

It is generally recognized that sufficient sleep time for adolescents is 8 or more hours [1]. However, as social factors such as prevalent use of electronic games/mobile phones and intensification of academic competitions have the effect of curtailing sleep time, insufficient sleep among adolescents is currently a worldwide issue that is associated with adverse health and behavioral consequences [2,3]. From the viewpoint of an international comparison of adolescents’ sleep time, East Asian countries (Japan and Korea) have reported the shortest sleep time; on average approximately 1 to 2 h less than those in American, European and Australian countries [4,5].

In addition to the increase of insufficient sleep, napping appears to be common in adolescents. Cross-sectional investigations conducted in Asia [6,7], Europe [8,9], South and North America [10,11], and Australia [12] have reported that 20–50% of adolescents have a napping habit. Though the reason to take a nap is often to catch up on sleep lost during the school week [12], studies examining Japanese adolescents [7,13] have indicated that napping after returning from school on weekdays is associated with later bedtimes, which results in sleep curtailment and daytime impairments such as irritability, chronic fatigue, and frequent falling asleep. Those characteristics were recently reconfirmed by a national survey [14] that found that 12.5% of high school students in Japan often take naps after returning from school, 32.0% sometimes take naps, and higher napping frequency is associated with poor subjective condition in the morning. While the causal relationship between those adverse symptoms and napping behavior is unclear, nappers go to bed later than non-nappers [12]. Fukuda and Ishihara [7] have demonstrated the positive relationship between later napping time and delayed bedtime. Furthermore, Fukuda and Ishihara [13] have reported that both delayed bedtime and napping habit were associated with daytime impairments.

Considering the daily schedule of high school students, extracurricular activities play a major role in their lives. Students who attend extracurricular activities after school leave school later than those who do not belong to an extracurricular club. Therefore, it is plausible that the students who participate in extracurricular activities take naps later, go to bed later and, consequently, suffer from insufficient sleep. For the students who belong to athletic clubs, the effects of physical exercise may affect their sleep propensity. While having an exercise habit is known to improve sleep and to extend sleep duration [15,16], the present condition among Japanese adolescents is exercising without sufficient sleep. Under such conditions, an exercise-induced increase in sleep propensity may be insufficiently compensated for by short sleep time, possibly causing daytime sleep problems, which are considered to result in decreased academic performance. Taking those issues into account, the present study aimed to examine associations between evening naps and extracurricular activities with daytime sleep problems in Japanese high school students. 

While nationwide surveys on Japanese adolescents have been conducted to examine sleep-related issues [7,17,18,19], those studies have suggested significant associations between sleep problems and the students’ intention to enter university or vocational school. Additionally, most final year high school students who intend to enter university discontinue their club activities and begin devoting themselves to the university entrance examinations. A recent study in Germany using a large sample, aged between 0 and 25 years, has demonstrated that the age between 15 and 17 years shows a breakpoint characterized by the shortest nocturnal sleep time and the largest social jet lag (difference of nocturnal sleep phase between weekdays and weekends) [20]. With this in mind, we determined the population of our study as high school students in 10th and 11th grade (from 15 to 17 years) who mostly intended on entering university. Further, although previous studies to examine sleep problems used a single criterion to identify excessive daytime sleepiness, which were different among the studies [6,8,9,18,19], we prepared two questions for identifying practical severity of daytime sleepiness. Those were to ask about: (1) excessive sleepiness during class; and (2) falling asleep during class, a starker symptom. In conjunction with a question asking about subjective insufficient sleep, which has been used in previous studies [6,18,19], three questions in total were prepared to evaluate daytime sleep problems.

Based on the present study population of high school students in 10th and 11th grade who mostly intended to enter university, the following two hypotheses were proposed for this study:

(1) Among students with smaller amounts of sleep, the students belonging to athletic clubs reveal a stronger association with daytime sleep problems compared to those belonging to cultural clubs and those with no club activity.

(2) An after-school napping habit is associated with delayed bedtime, shortened sleep time, and consequently daytime sleep problems.

## 2. Results

### 2.1. Sample Characteristics 

Table 1 presents the demographic and life habit variables of the students. In this group of students who mostly intended to enter university, 20.2% and 31.1% napped on weekdays after school ≥3 days/week and 1–2 days/week, respectively. With regard to participation in extracurricular club activities, 58.9% and 26.3% belonged to an athletic and a cultural club, respectively, and the students who did not belong to extracurricular club were in the minority (14.8%). 

### 2.2. Sleep Habits, Sleep Health, and Sleep Problems

Table S1 (see supplementary material) shows the results of sleep habits for each answer category of the demographic and life habit variables. For the respondents as a whole, the mean bedtime and rise time on weekdays were 23:52 ± 0:56 and 6:22 ± 0:41, respectively, resulting in a mean time in bed (TIB) of 390 ± 56 min. Selected attributes other than sex, one-way commute time and time spent studying and watching TV revealed a significant effect on weekday bedtime. The attributes showing remarkable differences of approximately 30 min among answer categories were weekday napping behavior, time spent using electronic devices on weekdays, and the frequency of using electronic devices within 30 min before bedtime. The more frequent or the longer answer categories showed later bedtimes. 

The attributes other than weekdays napping behavior and club activity demonstrated a significant effect on weekday rise times. The largest effect on weekdays rise time among the attributes was one-way commute time, showing that the students whose commute time was >1 h but ≤2 h got up 48 min earlier on average than those whose commute time was ≤30 min. With regard to TIB on weekdays, attributes other than skipping breakfast, and the time spent watching TV and using electronic devices on weekdays demonstrated significant effects. The attributes revealing the largest effect were one-way commute time and weekday napping behavior, demonstrating shorter TIB in the answer categories of longer commute time and more frequent napping after school, respectively. 

On weekends, for the respondents as a whole, mean rise time was approximately 1.5 h later than that on weekdays, resulting in a mean TIB of 466 ± 85 min, which was 76 min longer than that on weekdays. It should be noted that the questions used for four attributes (an after-school napping habit, times spent for studying, watching TV, and using electronic devices) asked the life habits on weekdays. Therefore, while causal associations between weekdays life habits and weekends sleep habits were unclear, the results of weekends sleep habits in those four attributes are considered as the characteristics of the students having those weekdays life habits. Nevertheless, the effect of each attribute on weekend sleep habits was mostly similar to that on weekdays. Notable findings to show different characteristics from those on weekdays were that rise time in the group who belonged to no club activity was approximately one hour later than those in the groups who belonged to athletic and cultural clubs, and that one-way commute time demonstrated no significant effect on TIB.

Prevalence of weekend oversleeping among the whole group of respondents was 17.4%. The results of the χ^2^ test showed a significant effect of weekday napping behavior, club activity, one-way commute time, time spent studying and using electronic devices on weekdays, and the frequency of using electronic devices within 30 min before bedtime. Among the answer categories of those attributes, the group who belonged to no club activity had the highest prevalence of weekend oversleeping (34.5%). The lowest prevalence (8.1%) was shown in the group whose time studying was >2 h on weekdays. 

Table S2 (see supplementary material) shows the results of the sleep health scores for each answer category of the demographic and life habit variables. For the respondents as a whole, the lowest T-score among the five sleep health factors was rising in the morning (54.4 ± 8.7). The answer categories revealing the top three lowest scores of rising in the morning were found in the categories of skipping breakfast ≥3 days/week (49.4 ± 10.6) and 1–2 days/week (51.4 ± 10.2), and taking naps after school ≥3 days/week (51.7 ± 9.7). As for the score of parasomnia, which was the second lowest as a whole (56.0 ± 7.3), skipping breakfast 1–2 days/week was the answer category revealing the lowest score (53.1 ± 9.3). The other three factor scores (respiration, sleep maintenance, sleep initiation) showed the whole average values around 60, and the average values of each answer category ranged between 57.4 ± 9.3 and 62.5 ± 6.5.

Table S3 (see supplementary material) presents the prevalence of sleep problems for each answer category of the demographic and life habit variables. For the respondents as a whole, prevalence of subjective insufficient sleep (SIS), excessive sleepiness during class (ESC), and falling asleep during class (FAC) were 63.9%, 55.1%, and 32.6%, respectively. Weekday napping behavior and time spent studying on weekdays were the attributes revealing significant differences among answer categories for all three sleep problems. Higher prevalence of sleep problems was shown with frequent napping behavior and shorter time spent studying. Although FAC was asked as a more severe problem than ESC, grade and club activity were the attributes revealing a significant difference only for FAC. Prevalence of FAC was higher among those in the 11th grade, and in those participating in cultural/athletic club activities. 

### 2.3. Characteristics of Napping Behavior

Table 2 shows the timing and duration of weekday napping behavior for each answer category of frequency of napping per week and club activity. For the respondents as a whole, the mean time to take naps was 19:54 ± 1:41, and 40.6% (the majority) of the respondents answered that they took naps for >30 min but ≤1 h. The respondents who took weekday naps ≥ 3 days/week began napping later and longer compared with those taking naps 1–2 days/week. The timing of taking naps among the respondents who did not belong to any clubs was more than 80 min earlier on average than those belonging to cultural or athletic clubs. The napping duration in each attribute of club activity showed no clear characteristics, whereas those belonging to no club revealed the highest and the lowest percentages to answer >2 h and ≤30 min, respectively. 

### 2.4. Association between Sleep Problems and Demographic and Life Habit Variables

Table 3 shows the results of the logistic regression analyses that were used to estimate the association between SIS and the demographic and life habit variables. Multiple logistic regression analyses revealed that a higher risk SIS was associated with female sex, one-way commute time ≥1 h, TIB <6 h on weekdays, oversleeping ≥3 h on weekends, and lower score of rising in the morning. On the other hand, TIB ≥7 h on weekdays was associated with a lower risk of SIS.

The results of the logistic regression analysis to examine the associated factors with ESC and FAC are shown in Table 4 and Table 5. The results of multiple logistic regression analyses revealed that some factors were associated with both problems, and that some factors were associated with only one of the problems. The factors associated with both problems were napping ≥3 days/week, belonging to an athletic club, frequently using electronic devices within 30 min before bedtime, oversleeping on weekends ≥3 h, and a lower score of rising in the morning. Female sex and a lower parasomnia score were associated with higher risk of ESC only. Being in the 11th grade, belonging to a cultural club, having a lower score of respiration, and having a higher score of sleep initiation were associated with a higher risk of FAC only. On the other hand, studying >1 h on weekdays and TIB ≥7 h on weekdays were associated with a lower risk of FAC. Among the attributes, the strongest associations with any of the three sleep problems were belonging to a cultural and an athletic club with a higher risk of FAC (adjusted odds ratio: 2.39 and 2.27, respectively). 

## 3. Discussion

The present study examined the association of students’ involvement in athletic and cultural clubs with daytime sleep problems. Although we hypothesized that the students belonging to athletic clubs would reveal a stronger association with daytime sleep problems compared to those belonging to cultural clubs or no club, the results of multiple logistic regression analyses supported the hypothesis only a slightly. Among the three variables of daytime sleep problems, only ESC showed a significant association specific to the athletic club students. As for SIS and FAC, the former revealed no significant association with extracurricular club activities, and the latter displayed a similar adjusted odds ratio for both the athletic and cultural club students. Although the reason that the athletic club students did not reveal a significant association with SIS is unclear, one fundamental background, which might affect subjective sleep evaluation, is short TIB on weekdays (6.5 h on average). Because the differences in TIB on weekdays among the extracurricular activity categories were small (mean TIB of 383 min, 381 min, and 395 min for no club activity, cultural club, and athletic club, respectively), there seems to be almost no difference in the degree of weekdays cumulative sleep loss among those three groups. As it has been experimentally confirmed that cumulative sleep loss induces an underestimation of subjective sleepiness compared to decreased objective vigilance performance [21,22], the question asking SIS might be vague and/or insensitive for detecting the effect of athletic activities under short TIB. On the other hand, questions asking frequency of excessive sleepiness (ESC) and falling asleep (FAC) during class might more precisely detect their daytime sleep problems. 

A notable finding was that the cultural club students showed a significant association with FAC. Their adjusted odds ratio of 2.39 was similar to that of the athletic club students (2.27). Those values were the highest among the attributes of any of the three sleep problems. As FAC was considered to be a more serious sleep problem than ESC from an academic point of view, the cultural club students as well as the athletic club students could suffer from unfavorable academic consequences. Possible reasons for the strong association between the cultural club students and FAC might be that activities in some cultural clubs, such as the brass band club, can last a long time and may include physical activities. Besides, among the athletic clubs, exercise type, duration, and intensity can vary considerably. Therefore, future studies should investigate detailed information of the club type and activities, not only for the cultural clubs but also for the athletic clubs.

Nationwide surveys on sleep problems in Japanese adolescents were published in 2004 [18] and 2010 [19]. Both studies examined the association of extracurricular activities with excessive daytime sleepiness but did not separately examine the association of athletic and cultural clubs. The former study found a significant association demonstrating the reduced risk of excessive daytime sleepiness (answering “always” or “often” to the question regarding excessive sleepiness during the daytime) among the students who actively participated in extracurricular activities, compared to the students who did not participate or participated, but not actively. In contrast, the latter study showed that the risk for excessive daytime sleepiness determined by the results of the Epworth sleepiness scale was higher among the students who participated in extracurricular activities. While the reason for the discrepancy is unknown, the adjusted odds ratios, which range from 1.07 to 1.15, are lower than those in the present study. Considering that those nationwide studies covered six grades, from 12 to 18 years old, the stronger odds ratio observed in the present study might be ascribed to the study sample; high school students in 10th and 11th grade who went to university-bound high schools.

Whereas having an exercise habit has been reported to be associated with improvement in sleep maintenance and extension of sleep duration [15,16], the present results of the athletic club students did not fully support those previous findings. As for sleep maintenance score, no significant group effect was detected, revealing high sleep maintenance scores in all three subject groups (Table S2). This suggested good sleep maintenance due to younger age and short TIB (around 6.5 h), resulting in there being no room for improvement by exercise habit in the athletic club students.

The second hypothesis for the association of an after school napping habit with nocturnal sleep time and daytime sleep problems is supported by the results (except for no association between the napping habit and SIS). The students who took an evening nap ≥3 days/week revealed delayed bedtimes, shorter TIB, and significantly higher risks for ESC and FAC. Consistent with the present findings, previous studies have indicated that adolescents’ napping behavior is associated with unfavorable symptoms, such as later bedtime and shorter sleep duration [7,10,12], difficulty in getting up in the morning, drowsiness during class, chronic fatigue, and irritability [8,10,13]. Causality between napping behavior and those symptoms has not been proven. Gradisar et al. [12] suggested that adolescents’ napping behaviors are compensative measures to catch up on sleep debt during weekdays. On the other hand, Fukuda and Ishihara [7] suggested that evening naps might induce a later bedtime, but that a later bedtime may not be the cause for evening naps. Further, Rahafar et al. [23] investigated sleep habits of Iranian people (aged 25.2 years on average), and found a positive correlation between midpoint of napping and midpoint of nocturnal sleep, but no correlation between nap duration and midpoint of nocturnal sleep. They suggested that longer naps do not seem to compensate for lack of sleep. 

In the present study, the timing of taking naps after school was 19:54 ± 1:41, with later timings indicated by the students belonging to cultural or athletic clubs. Two previous studies [7,12] reporting adolescents’ napping behavior, and the time at which naps were taken, indicated earlier napping times than those in the present study. Gradisar et al. [12] reported that Australian high school students took naps around 16:00, and Fukuda and Ishihara [7] reported that 35.6% of Japanese high school students took naps later than 17:00. The delayed napping time in the present study may be because the participants were only asked about napping behavior on the weekdays after returning home and not on the weekends. The timing from 2 to 4 h before habitual bedtime is known as “forbidden zone for sleep” when sleep propensity is at the lowest point during the circadian rhythm [24]. The reasons or mechanisms by which the present study sample could have a nap around the time of the “forbidden zone for sleep” were obscure. It’s possible that those who took such evening naps might be under the condition of vicious circle of taking evening naps, delaying the bedtime, shortening nocturnal sleep, and increasing daytime sleep problems. It is desirable to conduct a future study to examine the effects of napping cessation in the students whom habitually take evening naps after class.

As for duration of the naps, “>30 min but ≤60 min” was the largest category; answered by 40.6% of the nappers. According to a nationwide survey conducted on Japanese adolescents [19], the most common duration of the naps on weekdays was “≥15 min but <30 min,” answered by slightly less than 40% of the nappers in the same grade as the present study. However, as the question used in that study covered naps on the train/bus, the results are not comparable to the present study, which asked about the duration of naps after returning home only. In the case of having evening naps longer than 30 min, it may be possible that the students fell into slow-wave sleep to reduce homeostatic sleep pressure, which could possibly result in delayed bed time. As sleep progression and quality are affected by environmental conditions such as light [25,26], noise [27,28], bedding [29,30], and body position [31], future studies should ask about those conditions during the evening naps with subjective sleep evaluation.

The other notable attributes associated with daytime sleep problems were grade, study duration after returning home on weekdays, and frequency of using electronic devices within 30 min before bedtime. The adjusted odds ratio for the risks of FAC in the 11th grade was 2.08 compared with that in the 10th grade, which was much stronger than the previous nationwide study on Japanese adolescents [19], demonstrating almost no difference between those two grades (1.92 and 1.82, respectively, compared to that at 7th grade of junior high school). Because the TIBs on weekdays were almost the same between those grades (394 min and 385 min at 10th and 11th grade, respectively), other reasons, such as negligent habituation, may be related to the high risk in the 11th grade, which is considered to be a matter of grave concern for going on to university. The students who studied for 1 h or longer after returning home had significantly lower adjusted odds ratios for FAC. This may suggest that those students had more motivation for studying and/or the preparations to follow classes, preventing boredom during class. With regard to the association between electronic device use and daytime sleep problems, significant associations with ESC and FAC were detected only for frequency of using the devices within 30 min before bedtime, but not for total time of using devices. In conjunction with the findings of the Japanese nationwide survey [32], the present study supports the idea that electronic device use around bedtime is associated with sleep problems in Japanese adolescents.

In conclusion, the present study examined the association of napping after returning from school and extracurricular activities with daytime sleep problems in Japanese adolescents who mostly intended to enter university. Among the three daytime sleep problems, including subjectively insufficient sleep, excessive sleepiness during class, and falling asleep during class, the most severe, falling asleep during class ≥3 days/week, was answered by approximately one-third of the students. The three top associated factors for this daytime sleep problem are belonging to cultural clubs, belonging to athletic clubs, and taking evening naps ≥3 days/week, revealing an adjusted odds ratios above 2.0. Considering the possibility that the academic performance of those students will suffer because of this, appropriate measures should be provided to ensure sufficient sleep and to keep alert during class. Despite the relatively small sample size collected in a local area, the results focusing on the 10th and 11th grade university-bound high schools could be significant in considering their life habits and daily scheduling. Future studies are required to investigate larger sample sizes, including not only other locations in Japan but also the other countries, and to examine the effects of interventions to extend sleep duration and prohibit the habit of napping after returning from school.

## 4. Materials and Methods 

### 4.1. Subjects and Procedures

Previous surveys [7,17] of Japanese adolescents have demonstrated that insomniac symptoms and bedtime are associated with the intention to enter university or attending the type of school in which students are bound for university or a vocation. Therefore, we determined the study population as ordinary high school students who mostly intended to enter university. Also, during that year (2011), East Japan had suffered from the greatest earthquake in Japanese history. Taking those situations into account, we selected high schools where the academic level of the school was between the top 16th and 31st percentile and had not been damaged by the earthquake. Consequently, three prefectural high schools in Miyagi Prefecture were selected to participate in the present study. 

In the Japanese education system, the school term begins in April, and summer vacation, which last approximately 1 month, begins in the last part of July. After summer vacation, most of the students in the highest grade (12th grade) discontinue their club activities and begin devoting themselves to the university entrance examination. Also, sleep disturbance due to the hot and humid environment of the summer in Miyagi Prefecture is mostly relieved after the summer vacation. Therefore, we determined it was most appropriate to conduct the survey with the students in 10th and 11th grade (from 15 to 17 years) after summer vacation (early autumn). As the questionnaire adopted asked about symptoms and conditions of the subjects looking back over the previous month, the survey was conducted 4 to 5 weeks after summer vacation. A total of 1513 students were invited to the survey and 1314 individuals (743 females and 571 males) completed the questionnaire, which resulted in an 86.8% response rate.

This study was approved by the ethics committee of Tohoku Fukushi University (project identification code: RS1107195), and was conducted in accordance with the Declaration of Helsinki.

### 4.2. Measures

The questionnaire covered the following items: (1) demographic variables; (2) lifestyle habits; and (3) sleep habits and sleep-related issues. Questions regarding symptoms or conditions were for the previous month. Questions for demographic information included grade, age, and sex. Extracurricular club activities that a student participated in more than 3 days/week were assessed based on the following four answer categories: 1 = none, 2 = athletic club, 3 = cultural club, 4 = other, such as student council. Variables of lifestyle habits were determined by referring to previous studies demonstrating the significant associations of short nocturnal sleep duration and/or excessive daytime sleepiness in Japanese adolescents. Those were: skipping breakfast [18], commuting time [19,33], time spent studying [33], and using electronic devices such as electronic games, personal computers, or mobile phones [32]. Although time spent watching TV was reported not to be associated with short nocturnal sleep in Japanese high school students [33], we included this variable because this study [33] showed that time for watching TV was longer than that for studying and playing personal computer games. Each of the questions asking time spent studying, using electronic devices, and watching TV contained the four following answer categories: 1 = <30 min, 2 = ≥30 min but <60 min, 3 = ≥1 h but <2 h, 4 = ≥2 h, in the case of both weekdays and weekends. The same answer categories were used for asking commute time. Regarding the use of electronic devices, frequency of use within 30 min before bedtime was assessed based on the following four answer categories: 1 = almost never, 2 = rarely, 3 = sometimes, 4 = frequently. Additionally, frequency of skipping breakfast (1 = almost never, 2 = 1–2 days/week, 3 = 3 days or more/week) was assessed. 

Regarding the items on sleep habits, bedtime and rise time on weekdays and weekends were included. Based on those results, times from bedtime to rise time (time in bed (TIB)) were calculated. As a recent paper demonstrated that social jet lag for 16-year-olds is about 3 h [20], we defined a student whose rise time on weekends was delayed 3 h or more than on weekdays as a “weekend oversleeper”. Weekday napping behavior after returning home was investigated by asking questions about the following three aspects: frequency (1 = almost never, 2 = 1-2 days/week, 3 = ≥3 days/week); time (asked about nap start times); and duration (1 = ≤30 min, 2 = >30 min but ≤60 min, 3 = >1 h but ≤2 h, 4 = >2 h). Items on daytime sleep problems included the following three aspects: feeling subjective insufficient sleep (1 = too much sleep, 2 = satisfactory, 3 = uncertain, 4 = insufficient), feeling excessive sleepiness during class (1 = almost never, 2 = 1–2 days/week, 3 = 3 days or more/week), and falling asleep during class (1 = almost never, 2 = 1–2 days/week, 3 = 3 days or more/week). The answers, “insufficient” and “3 days or more” were taken as affirmative answers to respective questions to be identified as having sleep problems (subjectively insufficient sleep (SIS), excessive sleepiness during class (ESC), falling asleep during class (FAC)). A sleep health questionnaire (SHQ) was administered to the students to identify subjective sleep quality. The SHQ contained 14 questions that were used to calculate the T-scores for the five sleep health factors, consisting of sleep maintenance, parasomnia, respiration, sleep initiation, and rising in the morning. While the original version of the SHQ calculates factor scores of sleep health risk index scores, we transformed the scores to sleep health index scores; accordingly, the higher the score the better sleep health was considered to be. Detailed items of the questions and the method for calculating factor scores are introduced by Tanaka and Shirakawa [34]. Although the high school students were likely to give valid answers to those 14 questions, it should be noted that calculated T-scores are based on a database of Japanese adults.

### 4.3. Statistical Analysis

Firstly, we calculated the mean values of nocturnal sleep habits (bedtime, rise time, and TIB on weekdays and weekends) and the percentages with 95% confidence intervals (CIs) of weekend oversleepers for each answer category of the following attributes: sex; grade; frequency of weekday napping behavior; extracurricular activities; frequency of skipping breakfast; one-way commute time; time spent studying, watching TV, and using electronic devices on weekdays; and frequency of electronic device use within 30 min before bedtime. Similar calculations were conducted for the T-scores of the SHQ. Secondly, as for the results of sleep problems (SIS, ESC, FAC), percentages and 95% CIs of the students who gave affirmative answers for each answer category of the attributes described above were calculated. Thirdly, mean values of the time that the students take naps after returning home and percentages of the answer categories for napping durations (four categories described above) were calculated for each of the extracurricular activity categories and weekday napping behavior categories, respectively. To examine the statistical significance of the results, we used analysis of variance for continuous numerical data and the χ^2^ test for categorical data. Finally, a series of logistic regression analyses were performed in order to examine the association between demographic, lifestyle habits, sleep habits variables, and SHQ scores with the presence of each sleep problem. Detailed independent variables are shown in Tables presenting logistic regression results. With regard to the variables of sleep habits, TIB on weekdays (<6 h, ≥6 h but <7 h, ≥7 h), TIB on weekends (<7 h, ≥7 h but <8 h, ≥8 h), and oversleeping on weekends (<3 h, ≥3 h) were classified for analyses. Initially, each independent variable was examined using the univariate logistic model. Subsequently, the variables with *p* < 0.1 were adopted for multiple logistic regression analyses. As we focused on the association of extracurricular activities and weekday napping behavior with the presence of sleep problems, those were treated as forced entry variables in the multiple logistic regression model. The other variables were examined by using forward selection with removal testing based on the probability of the Wald statistic. Those statistical analyses were conducted using PASW Statistics 17.0.2 (IBM Corporation, Armonk, NY, USA). The level of significance was considered to be *p* < 0.05. Data are shown as mean ± standard deviation (SD).

## Figures and Tables

**Table 1:** Demographic and life habit variables of the participants.

	*N*	% or Years (SD ^1^)
Overall	1314	100.0
Age	1314	16.0 (0.7)
Sex		
Female	743	56.5
Male	571	43.5
Grade		
10th	672	51.1
11th	642	48.9
Napping behavior after returning home on weekdays		
None	640	48.7
1–2 days/week	408	31.1
≥3 days/week	266	20.2
Club activity		
None	194	14.8
Cultural club	346	26.3
Athletic club	774	58.9
Skipping breakfast		
None	1193	90.8
1–2 days/week	67	5.2
≥3 days/week	52	4.0
One-way commute time		
<30 min	679	52.1
≥30 min but <1 h	478	36.7
≥1 h but <2 h	147	11.3
Time spent studying on weekdays		
<30 min	190	14.5
≥30 min but <1 h	462	35.3
≥1 h but <2 h	544	41.6
≥2 h	111	8.5
Time spent watching TV on weekdays		
<30 min	309	23.6
≥30 min but <1 h	450	34.4
≥1 h but <2 h	415	31.8
≥2 h	133	10.2
Time spent using electronic devices such as mobile phones, electronic games, and personal computers on weekdays		
<30 min	257	19.6
≥30 min but <1 h	397	30.3
≥1 h but <2 h	397	30.3
≥2 h	258	19.7
Frequency of using electronic devices within 30 min before bedtime		
Almost never	153	11.7
Rarely	243	18.6
Sometimes	262	20.0
Frequently	650	49.7

^1^ SD, standard deviation.

**Table 2:** Timing and duration of napping behavior for each club activity and napping frequency category.

			Time		Duration				
		*N* (%)	mean (SD)	*p*-Value ^†^	≤30 min (%)	>30 min but ≤1 h (%)	>1 but ≤2 h (%)	>2 h (%)	*p*-value ^‡^
Overall		674 (100)	19:54 (1:41)		24.5	40.6	25.7	9.3	
				0.002					<0.001
Napping behavior after returning home on weekdays	1–2 days/week	408 (60.5)	19:45 (1:38)		28.0	44.8	22.6	4.6	
	≥3 days/week	266 (39.5)	20:09 (1:40)		19.3	35.1	29.7	15.8	
				<0.001					0.005
Club activity	None	110 (16.3)	18:38 (1:44)		15.7	47.2	20.4	16.7	
	Cultural club	171 (25.4)	20:01 (1:36)		28.1	42.5	21.0	8.4	
	Athletic club	393 (58.3)	20:14 (1:29)		25.4	38.4	29.1	7.1	

^†^ Analysis of variance. ^‡^ χ^2^ test.

**Table 3:** Logistic regression results for prediction of subjectively insufficient sleep.

		Univariate Model				Multivariate Model			
		*N*	Crude Odds Ratio	95% CI	*p*-Value	*N*	Adjusted Odds Ratio	95% CI	*p*-Value
Sex	Female	571	1.00 (ref)			558	1.00 (ref)		
	Male	741	1.63	1.30–2.05	<0.001	729	1.60	1.23–2.06	<0.001
Grade	10th	672	1.00 (ref)						
	11th	642	0.871	0.67–1.09	0.230				n.a.
Napping behavior after returning home on weekdays	None	638	1.00 (ref)			628			
	1–2 days/week	408	1.13	0.87–1.46	0.368	397			n.s.
	≥3 days/week	266	1.73	1.26–2.38	<0.001	262			n.s.
Club activity	None	193	1.00 (ref)			190			
	Cultural club	346	1.06	0.73–1.54	0.757	337			n.s.
	Athletic club	773	0.96	0.69–1.34	0.825	760			n.s.
Skipping breakfast	None	1189	1.00 (ref)						
	1–2 days/week	68	0.96	0.58–1.60	0.877				n.a.
	≥3 days/week	53	1.67	0.88–3.17	0.119				n.a.
One-way commute time	<30 min	678	1.00 (ref)			668			
	≥30 min but <1 h	476	1.28	1.00–1.64	0.050	469			n.s.
	≥1 but <2 h	152	2.49	1.63–3.79	<0.001	150	1.69	1.10–2.59	0.017
Time spent studying on weekdays	<30 min	191	1.00 (ref)			189			
	≥30 min but <1 h	462	0.73	0.50–1.06	0.095	454			n.s.
	≥1 but <2 h	542	0.59	0.41–0.85	0.004	535			n.s.
	≥2 h	111	0.53	0.32–0.87	0.013	109			n.s.
Time spent watching TV on weekdays	<30 min	308	1.00 (ref)						
	≥30 min but <1 h	450	0.85	0.62–1.15	0.288				n.a.
	≥1 but <2 h	414	0.80	0.59–1.09	0.161				n.a.
	≥2 h	133	0.94	0.61–1.45	0.776				n.a.
Time spent using electronic devices such as mobile phones, electronic games, and personal computers on weekdays	<30 min	256	1.00 (ref)						
	≥30 min but <1 h	397	1.05	0.76–1.45	0.768				n.a.
	≥1 but <2 h	397	1.27	0.91–1.76	0.157				n.a.
	≥2 h	258	1.24	0.86–1.78	0.245				n.a.
Frequency of using electronic devices within 30 min before bedtime	Almost never	153	1.00 (ref)			152			
	Rarely	244	1.23	0.81–1.85	0.327	242			n.a.
	Sometimes	261	1.31	0.87–1.97	0.194	257			n.a.
	Frequently	648	1.58	1.10–2.26	0.013	636			n.s.
TIB on weekdays	<6 h	262	2.35	1.64–3.39	<0.001	256	2.12	1.46–3.07	<0.001
	≥6 h but <7 h	540	1.00 (ref)			537	1.00 (ref)		
	≥7 h	507	0.58	0.45–0.75	<0.001	494	0.61	0.48–0.80	<0.001
TIB on weekends	<7 h	295	1.36	0.98–1.90	0.068	289			n.s.
	≥7 h but <8 h	341	1.00 (ref)			337			
	≥8 h	668	1.03	0.79–1.35	0.811	661			n.a.
Oversleeping on weekends	<3 h	1079	1.00 (ref)			1063	1.00 (ref)		
	≥3 h	228	1.82	1.32–2.52	<0.001	224	1.51	1.08–2.11	0.016
SHQ scores	Sleep maintenance	1312	0.99	0.97–1.01	0.378				n.a.
	Parasomnia	1312	0.99	0.97–1.00	0.078	1287			n.s.
	Respiration	1312	1.00	0.98–1.02	0.922				n.a.
	Sleep initiation	1312	0.99	0.98–1.01	0.380				n.a.
	Rising in the morning	1312	0.97	0.96–0.98	<0.001	1287	0.98	0.96–0.99	0.001

CI, confidence interval; TIB, time in bed; SHQ, sleep health questionnaire; n.a., not applicable for multivariate model due to *p* ≥ 0.1 by univariate logistic analysis; n.s., not significant.

**Table 4:** Logistic regression results for prediction of excessive sleepiness during class.

		Univariate Model				Multivariate Model			
		*N*	Crude Odds Ratio	95% CI	*p*-Value	*N*	Adjusted Odds Ratio	95% CI	*p*-Value
Sex	Female	572	1.00 (ref)			556			
	Male	745	1.24	1.00–1.54	0.055	728	1.29	1.01–1.66	0.043
Grade	10th	672	1.00 (ref)						
	11th	642	1.20	0.97–1.49	0.102				n.a.
Napping behavior after returning home on weekdays	None	641	1.00 (ref)			630	1.00 (ref)		
	1–2 days/week	409	1.36	1.06–1.75	0.015	397			n.s.
	≥3 days/week	267	2.67	1.96–3.63	<0.001	257	2.10	1.52–2.90	<0.001
Club activity	None	195	1.00 (ref)			187	1.00 (ref)		
	Cultural club	346	1.23	0.87–1.75	0.241	336			n.s.
	Athletic club	776	1.27	0.93–1.74	0.137	761	1.52	1.07–2.15	0.019
Skipping breakfast	None	1194	1.00 (ref)						
	1–2 days/week	68	1.25	0.76–2.07	0.374				n.a.
	≥3 days/week	53	1.00	0.57–1.73	0.994				n.a.
One-way commute time	<30 min	680	1.00 (ref)						
	≥30 min but <1 h	479	1.19	0.94–1.50	0.153				n.a.
	≥1 but <2 h	152	1.11	0.78–1.58	0.570				n.a.
Time spent studying on weekdays	<30 min	191	1.00 (ref)			184			
	≥30 min but <1 h	463	0.47	0.33–0.68	<0.001	451			n.s.
	≥1 h but <2 h	545	0.41	0.29–0.59	<0.001	540			n.s.
	≥2 h	111	0.29	0.18–0.48	<0.001	109			n.s.
Time spent watching TV on weekdays	<30 min	309	1.00 (ref)			303			
	≥30 min but <1 h	451	0.84	0.62–1.12	0.235	442			n.a.
	≥1 but <2 h	417	0.77	0.57–1.04	0.088	407			n.s.
	≥2 h	133	0.88	0.58–1.32	0.524	132			n.a.
Time spent using electronic devices such as mobile phones, electronic games, and personal computers on weekdays	<30 min	257	1.00 (ref)			254			
	≥30 min but <1 h	399	1.20	0.88–1.64	0.253	390			n.a.
	≥1 but <2 h	397	1.58	1.15–2.17	0.004	388			n.s.
	≥2 h	259	2.29	1.61–3.27	<0.001	252			n.s.
Frequency of using electronic devices within 30 min before bedtime	Almost never	153	1.00 (ref)			152			
	Rarely	245	1.06	0.71–1.59	0780	241			n.a.
	Sometimes	262	1.03	0.69–1.54	0.890	253			n.a.
	Frequently	651	2.21	1.55–3.16	<0.001	638	1.98	1.56–2.50	<0.001
TIB on weekdays	<6 h	264	1.42	1.05–1.93	0.023	255			n.s.
	≥6 h but <7 h	541	1.00 (ref)			535			
	≥7 h	509	0.74	0.58–0.94	0.013	494			n.s.
TIB on weekends	<7 h	297	1.47	1.07–2.01	0.017	290			n.s.
	≥7 h but <8 h	341	1.00 (ref)			335			
	≥8 h	671	1.10	0.85–1.43	0.480	659			n.a.
Oversleeping on weekends	<3 h	1083	1.00 (ref)			1062			
	≥3 h	229	1.81	1.34–2.44	<0.001	222	1.58	1.15–2.17	0.005
SHQ scores	Sleep maintenance	1317	0.99	0.97–1.01	0.158				n.a.
	Parasomnia	1317	0.97	0.95–0.98	<0.001	1284	0.98	0.96–0.99	0.008
	Respiration	1317	1.00	0.98–1.01	0.635				n.a.
	Sleep initiation	1317	1.00	0.99–1.02	0.910				n.a.
	Rising in the morning	1317	0.96	0.95–0.98	<0.001	1284	0.98	0.96–0.99	0.001

CI, confidence interval; TIB, time in bed; SHQ, sleep health questionnaire; n.a., not applicable for multivariate model due to *p* ≥ 0.1 by univariate logistic analysis; n.s., not significant.

**Table 5:** Logistic regression results for prediction of falling asleep during class.

		Univariate Model				Multivariate Model			
		*N*	Crude Odds Ratio	95% CI	*p*-Value	*N*	Adjusted Odds Ratio	95% CI	*p*-Value
Sex	Female	572	1.00 (ref)						
	Male	744	0.95	0.76–1.20	0.686				n.a.
Grade	10th	672	1.00 (ref)				1.00 (ref)		
	11th	642	2.08	1.64–2.63	<0.001		2.08	1.62–2.69	<0.001
Napping behavior after returning home on weekdays	None	641	1.00 (ref)			632	1.00 (ref)		
	1–2 days/week	409	1.05	0.80–1.38	0.737	398			n.s.
	≥3 days/week	266	2.03	1.51–2.72	<0.001	257	1.41	1.01–1.96	0.042
Club activity	None	195	1.00 (ref)			188	1.00 (ref)		
	Cultural club	345	2.07	1.37–3.12	<0.001	336	2.39	1.52–3.78	<0.001
	Athletic club	776	2.03	1.39–2.97	<0.001	763	2.27	1.49–3.46	<0.001
Skipping breakfast	None	1193	1.00 (ref)						
	1–2 days/week	68	1.28	0.78–2.13	0.330				n.a.
	≥3 days/week	53	0.61	0.32–1.17	0.135				n.a.
One-way commute time	<30 min	679	1.00 (ref)						
	≥30 min but <1 h	479	0.95	0.74–1.23	0.713				n.a.
	≥1 but <2 h	152	1.18	0.81–1.70	0.390				n.a.
Time spent studying on weekdays	<30 min	191	1.00 (ref)			186	1.00 (ref)		
	≥30 min but <1 h	463	0.58	0.41–0.82	0.002	452			n.s.
	≥1 but <2 h	544	0.35	0.25–0.49	<0.001	540	0.60	0.46–0.79	<0.001
	≥2 h	111	0.30	0.18–0.51	<0.001	109	0.59	0.36–0.98	0.043
Time spent watching TV on weekdays	<30 min	309	1.00 (ref)			303			
	≥30 min but <1 h	450	0.79	0.58–1.07	0.120	442			n.a.
	≥1 but <2 h	417	0.67	0.49–0.91	0.011	410			n.s.
	≥2 h	133	0.95	0.62–1.45	0.822	132			n.a.
Time spent using electronic devices such as mobile phones, electronic games, and personal computers on weekdays	<30 min	257	1.00 (ref)			254			
	≥30 min but <1 h	399	0.92	0.66–1.30	0.648	393			n.a.
	≥1 but <2 h	396	1.09	0.78–1.52	0.623	387			n.a.
	≥2 h	259	1.44	1.00–2.07	0.050	253			n.s.
Frequency of using electronic devices within 30 min before bedtime	Almost never	153	1.00 (ref)			152			
	Rarely	245	0.79	0.49–1.27	0.325	243			n.a.
	Sometimes	262	1.09	0.69–1.71	0.720	254			n.a.
	Frequently	650	1.99	1.34–2.95	<0.001	638	1.91	1.47–2.48	<0.001
TIB on weekdays	<6 h	263	1.46	1.08–1.98	0.014	256			n.s.
	≥6 h but <7 h	541	1.00 (ref)			536	1.00 (ref)		
	≥7 h	509	0.69	0.53–0.90	0.007	495	0.66	0.50–0.87	0.003
TIB on weekends	<7 h	296	1.31	0.95–1.82	0.101	290			n.a.
	≥7 h but <8 h	341	1.00 (ref)			335			
	≥8 h	671	0.93	0.70–1.23	0.611	662			n.a.
Oversleeping on weekends	<3 h	1082	1.00 (ref)			1065	1.00 (ref)		
	≥3 h	229	1.76	1.31–2.35	<0.001	222	1.63	1.18–2.25	0.003
SHQ scores	Sleep maintenance	1316	1.01	0.99–1.03	0.456				n.a.
	Parasomnia	1316	0.98	0.96–0.99	0.003	1287			n.s.
	Respiration	1316	0.98	0.97–1.00	0.039	1287	0.98	0.96–1.00	0.032
	Sleep initiation	1316	1.01	1.00–1.03	0.084	1287	1.02	1.00–1.04	0.025
	Rising in the morning	1316	0.96	0.95–0.98	<0.001	1287	0.97	0.96–0.99	<0.001

CI, confidence interval; TIB, time in bed; SHQ, sleep health questionnaire; n.a., not applicable for multivariate model due to p≥0.1 by univariate logistic analysis; n.s., not significant.

## Supplementary Materials

The followings are available online at https://www.mdpi.com/2624-5175/1/3/30/s1, Table S1: Nocturnal sleep habit variables of the participants; Table S2: T-scores of five sleep health factors; Table S3: Daytime sleep problems among the participants.

## Abbreviations

TIB	Time in bed
SIS	Subjectively insufficient sleep
ESC	Excessive sleepiness during class
FAC	Falling asleep during class
SHQ	Sleep health questionnaire
CIs	Confidence intervals
